# Laryngeal tuberculosis without pulmonary involvement 

**Published:** 2012

**Authors:** Keyvan Kiakojuri, Mohammad Reza Hasanjani Roushan

**Affiliations:** 1 Department of Otolaryngology, Ayatollah Rouhani Hospital, Babol University of Medical Sciences, Babol, Iran.; 2 Infectious Diseases and Tropical Medicine Research Center, Babol University of Medical Sciences, Babol, Iran.

**Keywords:** Tuberculosis, Primary, Vocal cord, Hoarseness.

## Abstract

**Background:**

Tuberculosis of the larynx is a rare form of tuberculosis. Patients usually present with hoarseness or dysphagia and other nonspecific constitutional symptoms like fever or localized pain. In this study, we present a case of primary vocal cord lesion with tuberculosis.

**Case presentation:**

A 72 year old man presented with hoarseness of voice, low grade fever, and night sweating with in three month duration. Laryncoscopic study showed unilateral thickening of vocal cord and biopsy of the lesion showed granuloma with caseous necrosis. Chest x-ray was normal. The patient was treated with standard regimen of tuberculosis and was cured after 6 months of therapy.

**Conclusion:**

Laryngeal tuberculosis should be considered in the differential diagnosis of patients with hoarseness without pulmonary involvement in endemic regions of tuberculosis.

Laryngeal tuberculosis classically develops due to direct spread of mycobacterium tuberculosis to the larynx from contaminated sputum but can also occur due to hematogenous spread. Recently tuberculosis of larynx has often been diagnosed by clinicians attempting to rule out carcinoma (1). In preantibiotic era, laryngeal involvement was seen in more than one third of cases dying due to pulmonary tuberculosis. Incidence of laryngeal tuberculosis is less than 1% of all tuberculosis cases (2). At present, more than half of laryngeal tuberculosis cases are due to hematogenous seeding (3). 

Lesions vary from erythema to ulceration and masses resembling carcinoma (4). Direct laryngoscopy and biopsy are mandatory to establish a definitive diagnosis. It should be kept in mind that both tuberculosis and malignancy may coexist in the same patient (5). Diagnosis of laryngeal tuberculosis is made by identification of a caseating granuloma in a biopsy specimen. The patients respond well to antituberculosis drugs treatment. This case report describes primary laryngeal tuberculosis in a patient without pulmonary tuberculosis. 

## Case presentation

A 72 years old man presented to the Outpatient Clinic of Otolarygology of Rouhani Teaching Hospital with complaints of hoarseness, low grade fever with decreased appetite for a duration of one month. He had no cough or expectoration. The patient was not a smoker and with no alcohol and drug abuse. His wife had pulmonary tuberculosis 14 years ago and was treated with anti tuberculosis agents. She died five years ago due to myocardial infarction. His 14 year old daughter had subcutaneous tuberculosis of the femur 16 years ago without involvement of her lung or spine.

**Figure 1:**
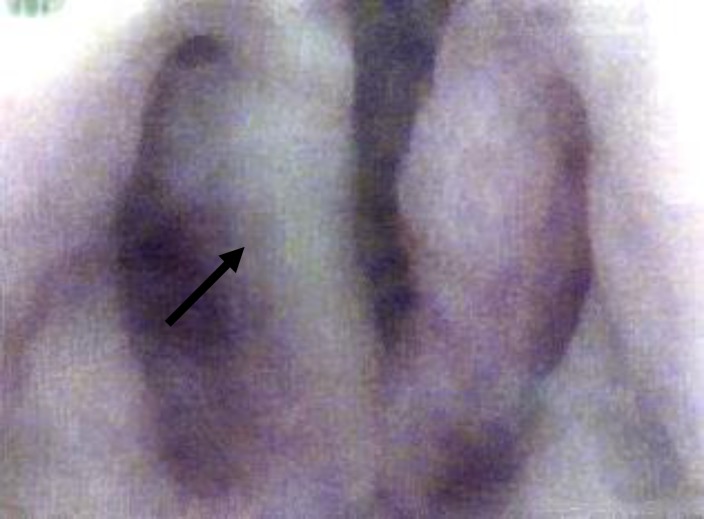
Vocal cord lesion at the time of presentation

In general physical examination, he was conscious. There was no cervical lymphadenopathy or clubbing. There were no scars or sinuses in the neck. Indirect laryngoscopy had shown a growth in the right ary-epiglottic fold (figure 1). Vocal cords were moving with no signs of infiltration. The respiratory system examination and chest x-ray were normal. PPD test showed 15 mm indurations after 48 hours. After a standard evaluation, the patient underwent laryngoscopy under local anesthesia and biopsy was taken from epiglottis.

**Figure 2:**
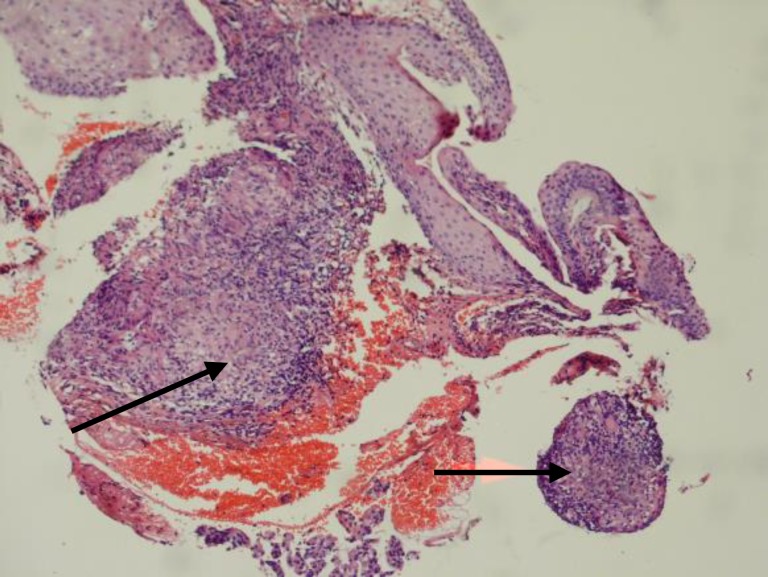
Pathologic examination of sample of the right vocal cord

The histopathological examination revealed biopsy tissue lined by stratified squamous epithelium showing focal dysplasia. Underlying stroma showed diffuse infiltration by lymphocytes, plasma cells, occasional polymorphs along with epitheloid granulomas, langhans giant cells and caseous necrosis (figure 2). Histopathologic findings confirmed tuberculosis as the cause of his hoarsness and he was treated with standard regimens of therapy.

**Figure 3:**
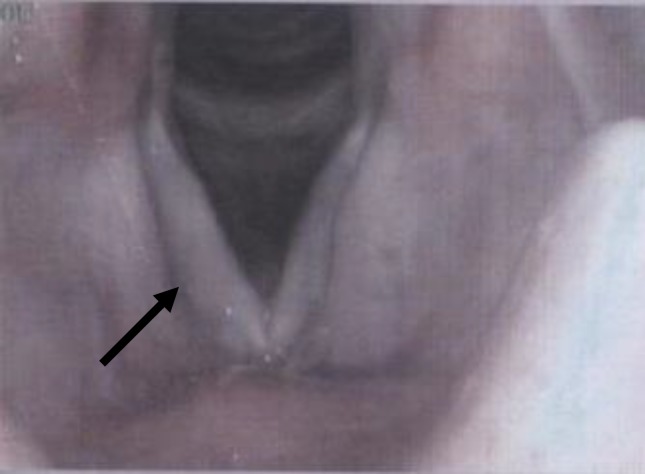
Vocal cord of the patient after treatment

A standard six month treatment with a combination of isoniazid, rifampicin, pyrazinamide, and ethambutol was started for two months followed by isoniazid and rifampicin for additional four months. The follow up after treatment showed resolution of the symptoms and improvement of the mass (figure 3).

## Discussion

Primary tuberculosis of larynx is rare. The accepted route of infection is direct invasion by inhaled tubercle bacilli. In this study, we present a case of vocal cord involvement due to mycobacterium tuberculosis without having any lesion in his lungs. Laryngeal tuberculosis is the most common granulomatous disease of the larynx and has usually been considered to result from pulmonary tuberculosis, although it might be localized in the larynx as a primary lesion without pulmonary involvement (6). The pathogenesis of laryngeal involvement is either primary or secondary (7, 8). Primary lesions occur in the absence of pulmonary disease. In the present case, the laryngeal involvement was probably a primary lesion due to contact with his wife who had pulmonary tuberculosis before. Early descriptions of laryngeal tuberculosis identified the posterior part of larynx as the part most frequently affected (9). In larynx, the commonest parts involved are the vocal cords and the least affected is the epiglottis (10). Laryngeal tuberculosis may be categorized to ulcerative lesions, nonspecific inflammatory lesions, polypoid lesions and ulcerofungative mass lesions (3, 11). 

In the present case, ulcerofungative mass lesion was present on the epiglottis. The patient responded to antituberculosis therapy by showing improvement in hoarseness of voice within three months. This case is a warning that a growth-like lesion in the upper respiratory tract could be tuberculosis in origin and, therefore, efforts should be made to locate an active or inactive lesion elsewhere in the body. Since there was no evidence of the disease in any other organ or system as evidenced by clinical, radiological examinations, the diagnosis of primary laryngeal tuberculosis was considered likely in our patient. Primary laryngeal tuberculosis was rarely reported in the medical literature (12-15). In conclusion, in endemic regions of tuberculosis any patient with hoarseness, tuberculosis should be considered in the differential diagnosis.

## References

[1] Loehrl TA, Smith TL (2001). Inflammatory and granulomatous lesions of the larynx and pharynx. Am J Med.

[2] Egeli E, Oghan F, Alper M, Harputluoglu U, Bulut I (2003). Epiglottic tuberculosis in patient treated with steroids for Addison's disease. Tohoku J Exp Med.

[3] Fitzgerald DW, Sterling TR, Hass DW, Mandell GL, Bennett JE, Dolin R (2010). Mycobacterium tuberculosis. Principles and Practice of Infectious Diseases.

[4] Lindell MM Jr, Jing BS, Wallace S (1977). Laryngeal tuberculosis. AJR AM J Roentgenol.

[5] Verma SK (2007). Laryngeal tuberculosis clinically similar to laryngeal cancer. Lung India.

[6] Kruschinski C, Welkoborsky HJ (2005). Tuberculosis of the larynx associated with orofacial granulomatosis in childhood. Otolaryngol Head Neck Surg.

[7] Jan A (1986). Primary laryngeal tuberculosis: a case report. J Laryngol Otol.

[8] Richter B, Fradis M, Kohler G, Ridder GJ (2001). Epiglottic tuberculosis: differential diagnosis and treatment Case report and review of literature.. Ann Otol Rhinol Laryngol.

[9] Horowitz G, Kaslow R, Friedland G (1976). Infectiousness of laryngeal tuberculosis. Am Rev Respir Dis.

[10] Fernandes L, Mesqnita A (1997). Stridor presentation in laryngeal tuberculosis. Indian J Tuberc.

[11] Shin JE, Nam SY, Yoo SJ, Kim SY (2000). Changing trends in clinical manifestations of laryngeal tuberculosis. Laryngoscope.

[12] Mehndiratta A, Bhat P, D’Costa L, Mesquita AM, Nadkarni N (1997). Primary tuberculosis of larynx. Indian J Tuberc.

[13] Baxi S, Jha S (2010). Primary laryngeal tuberculosis a rare entity. J Indian Med Assoc.

[14] Kozakiewicz J, Dec M, Gorczyca-Tarnowska J (2006). The rare case of primary isolated tuberculosis in a 19 year-old patient. Otolaryngol Pol.

[15] Edizer DT, Karaman E, Mercan H (2010). Primary tuberculosis involving epiglottis: a rare case report. Dysphagia.

